# Identifying Barriers in the Prescription Pathway of Special Low-Protein Foods and Protein Substitutes for People with PKU in the UK: An Industry Perspective

**DOI:** 10.3390/nu18183095

**Published:** 2026-09-21

**Authors:** Martina Tosi, Suzanne Ford, Anne Grimsley, Sarah Bailey, Anita MacDonald

**Affiliations:** 1 Department of Dietetics, Birmingham Women’s and Children’s Hospital, Birmingham B4 6NH, UK; martina.tosi@nhs.net; 2 National Society for Phenylketonuria (NSPKU), Sheffield S12 9ET, UK; suzanne.ford@nspku.org; 3 Dept of Nutrition & Dietetics, Bristol NHS Foundation Trust, Bristol Royal Infirmary, Bristol BS2 8HW, UK; 4 Royal Belfast Hospital for Sick Children, 274 Grosvenor Rd, Belfast BT12 6BA, UK; anne.grimsley@belfasttrust.hscni.net; 5 All Wales Inherited Metabolic Disease Service, University Hospital of Wales, Heath Park Way, Cardiff CF14 4XW, UK; sarah.bailey3@wales.nhs.uk

**Keywords:** phenylketonuria, special low-protein foods, protein substitutes, prescription, general practitioner, home delivery, pharmacy

## Abstract

**Background/Objectives**: Timely access to protein substitutes and special low-protein foods (SLPFs) is fundamental to maintaining dietary adherence and metabolic control in phenylketonuria (PKU). Disruptions within the prescription pathway may compromise the continuity of medical nutrition supply, with potential consequences for patient outcomes and health service efficiency. This study aimed to identify the most frequently reported prescription-related challenges from the perspective of UK industry stakeholders and to outline their implications for the care and dietetic practice of individuals with PKU. **Methods**: A structured questionnaire was distributed by the National Society for Phenylketonuria to industry representatives involved in supplying Advisory Committee on Borderline Substances (ACBS) approved protein substitutes and special low-protein foods (SPLFs). Anonymised responses were analysed descriptively to identify the types, frequency, and impact of prescription-related issues. **Results**: Forty-four prescribing and supply incidents were reported by specialist nutrition manufacturers and suppliers. Reported incidents affected both paediatric (54%) and adult (46%) patients, with protein substitutes accounting for the largest proportion of prescribing and supply issues (48%), followed by SLPFs (27%) and combined product issues (25%). Most reported barriers originated within primary care, particularly general practitioner (GP) practices (61%), followed by community pharmacies (21%). Thematic analysis identified five key areas of difficulty: GP prescribing barriers, prescribing errors, pharmacy and wholesaler procurement problems, supply chain and distribution challenges, and communication and care coordination failures. These challenges affected continuity of dietary treatment, increased the burden on patients, carers and healthcare professionals, generated additional financial costs, and highlighted wider inefficiencies within the prescribing and supply pathway. Fewer than half of the reported incidents were resolved (41%). **Conclusions**: Addressing these system-level barriers through improved prescribing processes, clearer communication pathways, and better coordination between stakeholders may enhance continuity of dietary treatment, reduce burden on patients and healthcare professionals, and support more efficient delivery of specialist nutritional care for individuals with PKU.

## 1. Introduction

Phenylketonuria (PKU) is a rare inherited metabolic disorder and for the majority of patients, lifelong dietary treatment remains central to management [1,2]. Maintaining blood phenylalanine (Phe) concentrations within recommended therapeutic ranges depends on continuous access to two essential components of treatment: protein substitutes, which provide zero or minimal Phe protein and usually contain added micronutrients, and special low-protein foods (SLPFs), which improve dietary adequacy, variety and long-term adherence [1,3,4]. These products are integral to nutritional therapy and must be available without interruption throughout life.

In the United Kingdom (UK), protein substitutes and SLPFs are prescribed through primary care but supplied through a complex network involving specialist metabolic dietitians, general practitioners (GPs), community pharmacies, prescribing support teams, manufacturers and home delivery providers [5,6]. Although the system is designed to ensure equitable access to specialist medical nutrition, multiple organisations and personnel contribute to each prescription, increasing the potential for delays, communication failures and supply errors [6]. The pathway is particularly challenging for SLPFs, where prescriptions require frequent adjustment to accommodate changing nutritional requirements, age, food preferences and clinical circumstances. In the UK, most prescriptions are issued on script for 30 days’ duration, and for patients with PKU, it is likely that the products needed will be supplied from more than one specialist dispenser. Thus, the number of scripts will be multiple: i.e., potentially up to 4 different scripts per month or 48 per year per patient from 4 different suppliers.

Interruptions to the supply of protein substitutes or SLPFs have important clinical consequences. Missed or delayed deliveries, prescribing inaccuracies and dispensing errors may reduce dietary adherence by increasing reliance on alternative higher protein foods and thereby compromising metabolic control and adversely affect quality of life [5]. These disruptions also generate considerable administrative workload for metabolic dietitians, who often spend substantial time resolving prescription problems rather than providing direct clinical care [7]. Consequently, inefficiencies within the prescribing pathway affect not only patients but also healthcare professionals and the organisations responsible for supplying medical nutrition [7].

Previous studies have largely examined prescribing challenges from the perspectives of patients and healthcare professionals, with limited attention given to the experiences of manufacturers and home delivery providers of medical foods [5,8]. However, these organisations are uniquely positioned to identify recurrent system failures, operational barriers and inefficiencies that may not be visible within clinical practice alone. Understanding these challenges is particularly timely as alternative prescribing models, including independent prescribing and centralised supply systems, are being considered across the UK.

This study aimed to characterise the challenges encountered by manufacturers and home delivery providers within the current UK prescribing pathway for protein substitutes and SLPFs, identify the principal causes of prescribing and supply failures, and explore their implications for patient care, service delivery and future redesign of prescribing systems.

## 2. Materials and Methods

### 2.1. Questionnaire Development

A structured questionnaire was developed to collect information on prescription pathway issues encountered during routine practice. It included eight questions, comprising five multiple-choice items and three free-text questions, designed to capture data on the type of issue, the products involved, the stage of the pathway affected, and the perceived impact on workflow and resources. The full questionnaire is provided in Table A1.

### 2.2. Questionnaire Distribution

The questionnaire was distributed by the National Society for Phenylketonuria (NSPKU) to representatives of manufacturers and home delivery providers involved in supplying Advisory Committee on Borderline Substances (ACBS) approved SLPFs and protein substitutes for individuals with PKU in the UK. Respondents were eligible to participate if they represented an organisation involved in the supply of protein substitutes and/or SLPFs for individuals with PKU in the UK. Data were collected between December 2025 and June 2026.

### 2.3. Data Analysis

Descriptive statistics were used to summarise quantitative data and are presented as frequencies and percentages. Respondents could report more than one incident, and therefore the number of reported incidents did not correspond to the number of individual respondents. Free-text responses were analysed using conventional content analysis to identify recurrent barriers within the prescribing and supply pathway. Responses were first reviewed to identify recurring issues and patterns, and initial codes were assigned to key issues identified within the responses. These codes were then grouped inductively into overarching themes and subthemes rather than being predefined. The resulting thematic structure was reviewed across responses to ensure consistency in the grouping of similar issues and to identify areas of overlap between related themes. Representative quotations were selected to illustrate the main barriers identified. The questionnaire did not collect patient identifiers or information that would allow multiple incidents to be reliably linked to the same patient or clinical pathway. Therefore, the reported incidents were not assumed to represent independent patient-level observations, and percentages describe the distribution of reported incidents rather than the prevalence of barriers among patients or prescriptions.

### 2.4. Ethics

This study was based on anonymised survey responses collected from industry representatives as part of a service evaluation of the PKU prescription and supply pathway. Participation was voluntary, and completion of the questionnaire was taken as consent to participate. No patient-level inclusion or exclusion criteria, including clinical characteristics, were applicable, as the study collected industry-level information rather than patient data. Ethical approval was not required, as this study involved anonymised data on prescription and supply issues reported by organisations involved in the provision of specialist nutritional products.

## 3. Results

A total of 44 prescribing and supply incidents were reported by representatives from five of the eight manufacturers and home delivery providers eligible to participate, supplying protein substitutes and SLPFs for people with PKU. Patient age was reported for 41 incidents, of which 54% (22/41) involved paediatric patients and 46% (19/41) involved adults. Protein substitutes were involved in 48% (21/44) of incidents, SLPFs in 27% (12/44), and both product types in 25% (11/44).

Among the 44 reported incidents, 61% (27/44) were ascribed by respondents to GP practices, followed by community pharmacies (21%, 9/44). Relatively few were attributed to home delivery providers (5%, 2/44), supplier stock shortages (2%, 1/44), or other isolated causes, including wholesaler issues, communication failures, and incorrect supplier allocation. Fewer than half of incidents were fully resolved (41%, 18/44), while 27% (12/44) remained unresolved; the remainder were partially resolved, ongoing, or of unknown outcome.

Five overarching themes were identified: (1) GP prescribing barriers, (2) prescribing errors, (3) pharmacy and wholesaler procurement problems, (4) supply chain and distribution challenges, and (5) communication and care coordination failures (Table 1).

### 3.1. GP Prescribing Barriers

GP-related prescribing barriers were the predominant cause of disruption to the supply of protein substitutes and SLPFs. Respondents described refusal by GP practices to accept repeat prescription requests from recognised third-party providers, including community pharmacies, dispensing appliance contractors, and specialist nutrition companies. Many practices instead required patients or carers to request prescriptions directly, increasing administrative burden and delaying patient access to treatment.

Administrative delays were common throughout the prescribing process, including prolonged authorisation times, unsigned or unprocessed prescriptions, and delays in electronic transmission. Several examples referred to prescriptions remaining outstanding for weeks or months, repeated telephone calls to GP practices before prescriptions were processed, unsigned prescriptions awaiting GP approval, and prescriptions reported as dispatched despite remaining within the practice. These often resulted in postponed deliveries, with some home delivery providers supplying products before receiving prescriptions to avoid interruptions to treatment, which is financially unsustainable.

Respondents also reported GP prescribing decisions that conflicted with recommendations from specialist metabolic teams, including reluctance to prescribe newly recommended products or implement dietary changes. Limited GP knowledge of PKU contributed to these problems, with some practices viewing protein substitutes and SLPFs as optional nutritional supplements, suggesting over-the-counter alternatives, or declining prescriptions because of perceived cost. Electronic prescribing system limitations included specialist products being unavailable on prescribing software, products being removed or inactive on repeat prescription lists, incorrect repeat prescriptions, and unsigned electronic prescriptions delaying prescription processing. Poor communication between GP practices, specialist teams, suppliers, and patients compounded these problems, creating uncertainty regarding prescription status and delaying implementation of treatment changes. The key themes identified relating to GP prescribing barriers are summarised in Table 1.

### 3.2. Prescribing Errors

Incorrect prescribing by GP practices was a recurrent barrier to the timely provision of protein substitutes and SLPFs. Errors affected both new and repeat prescriptions and included incorrect products, quantities, protein equivalents, dosages, and flavours. Under-prescribing was common, with prescribed quantities differing from those recommended by metabolic dietitians. Failure to update repeat prescriptions following changes in dietary management resulted in recurrent prescribing errors over successive prescription cycles despite repeated communication from specialist teams. Duplication of products within repeat prescribing systems, including multiple flavours or versions of the same product, also contributed to unnecessary prescribing and increased administrative burden. These findings were further supported by qualitative responses from manufacturers, who highlighted recurring issues related to prescription accuracy, communication gaps, and challenges within repeat prescribing systems. Representative quotes illustrating these barriers are presented in Table 2.

### 3.3. Community Pharmacy Procurement and Supply Chain Challenges

Many reported supply problems reflected failures in procurement processes rather than legitimate product shortages. Community pharmacies frequently relied on standard wholesaler ordering systems which incorrectly classified dietary products as unavailable despite established procurement routes. Electronic orders submitted by community pharmacies did not always transmit successfully to wholesalers, causing delays despite products being available. Further delays arose when community pharmacies postponed requests, failed to recognise that orders had not been processed, or unnecessarily returned prescriptions to GP practices after incorrectly concluding that items could not be sourced. Respondents reported that these problems often persisted despite previous education and support from manufacturers and wholesalers. A smaller number of incidents arose within the wider supply chain. These included wholesaler account restrictions, delivery errors, fragmented distribution arrangements, and discrepancies between perceived and actual product availability. These failures delayed product access despite adequate stock being available.

### 3.4. Communication and Care Coordination Failures

Communication and care coordination failures were a recurrent source of disruption across the prescribing and supply pathway. Respondents described poor information exchange between GP practices, community pharmacies, home delivery providers, specialist nutrition companies, and metabolic services, contributing to incorrect prescription routing, delays in implementing treatment changes, and fragmented patient care. Prescriptions were sometimes electronically routed to the wrong supplier or retained rather than promptly returned for reassignment, delaying access to essential products. Inadequate communication of dietary regimen changes also resulted in discontinued products continuing to be supplied because supplier records had not been updated. Respondents further highlighted the complexity created by multiple prescribing systems, separate supply routes for protein substitutes and SLPFs, and increasing use of centralised prescribing hubs and NHS App-based ordering, all of which increased the risk of communication failures and reduced coordination across the care pathway.

### 3.5. Impact of Prescribing and Supply Pathway Failures

Prescribing and supply pathway failures were reported to have potential clinical, operational, and financial consequences for patients, healthcare professionals, and supply organisations. Among the 44 incidents reported, delays in accessing protein substitutes and SLPFs frequently required emergency supplies or alternative arrangements to maintain treatment continuity. Respondents expressed concerns that prolonged disruption could compromise dietary adherence and metabolic control, particularly during clinically vulnerable periods such as pregnancy and transition to adult care. Notably, amongst specialist nutrition companies there is often direct communication with patients and families, so the industry respondents taking part in this project had direct knowledge of patient impact. Responses show that patients and families also experienced increased practical burden, anxiety, frustration, and reduced confidence in healthcare services as they were required to monitor prescription progress and coordinate resolution of supply problems.

Respondents described additional workload across metabolic services, GP practices, pharmacies, manufacturers, wholesalers, and home delivery providers. Resolving individual cases often required repeated communication, emergency product provision, specialist procurement support, and coordination between multiple organisations, diverting resources from routine clinical care and operational activities.

Respondents also reported significant financial consequences, including unreimbursed emergency supplies, additional courier and distribution costs, product wastage arising from prescribing errors, and inefficient use of healthcare and industry resources. Overall, respondents considered many of these problems to arise from fragmented prescribing systems, poor communication, and unclear organisational responsibilities, highlighting the need for greater integration and coordination across the specialist prescribing pathway. The key themes identified relating to the impact of prescribing and supply pathway problems are summarised in Table 3.

## 4. Discussion

This study provides the first reported industry perspective on prescribing and supply pathway barriers affecting access to protein substitutes and SLPFs for individuals with PKU in the UK. Although these specialist nutritional products are generally available, our findings indicate that interruptions in access are commonly attributable to failures in prescribing, procurement, communication, and coordination. Together, these findings highlight important vulnerabilities within the current pathway for delivering lifelong dietary treatment.

The high proportion of reported incidents attributed to GP practices highlights a potential mismatch between the organisation of primary care prescribing and the requirements of lifelong specialist metabolic management. These findings represent barriers as perceived and reported by suppliers and should not be interpreted as independently verified failures attributable to specific professional groups. Unlike conventional medicines, protein substitutes and SLPFs require regular review and adjustment according to metabolic control, age, growth, pregnancy, and dietary tolerance [2,9,10,11]. Consequently, prescribing relies on continuous collaboration between specialist metabolic teams and primary care. Similar challenges have been described across rare diseases, where fragmented care, limited disease-specific expertise, and poor integration between specialist and community services frequently compromise continuity of treatment [12,13,14]. Our findings suggest that the current prescribing model may place responsibility for highly specialised nutritional management within systems that are neither designed nor adequately supported to deliver it.

The recurrent prescribing errors identified in this study also suggest structural limitations within existing primary care prescribing systems. Errors were frequently repeated over successive prescription cycles despite intervention from specialist metabolic teams, indicating that repeat prescribing systems are not sufficiently responsive to regular dietary modifications. Unlike many long-term medications for general medicine, specialist nutritional prescriptions are dynamic and require frequent adjustment [2,9,15]. This raises questions about whether conventional community-based, repeat prescribing systems are appropriate for managing specialist nutritional therapies and if greater integration of specialist dietetic prescribing expertise is required.

Many reported supply problems reported by community pharmacies represented failures of the procurement pathway rather than failures of product supply. Community pharmacies frequently encountered difficulties accessing specialist nutritional products because conventional wholesaler ordering systems were poorly suited to low-volume specialist items. This distinction has important policy implications. Efforts to improve access should extend beyond ensuring adequate product availability to redesigning procurement pathways, improving interoperability between ordering systems, and strengthening coordination between pharmacies, wholesalers, manufacturers, and specialist providers [7,11]. Such system-level changes are likely to have a greater impact on reducing delays than focusing solely on stock availability.

Communication failures were another recurring feature and reflected the complexity of coordinating care across multiple organisations. Patients with PKU rely on GP practices, pharmacies, manufacturers, wholesalers, home delivery providers, and regional specialist metabolic centres, each operating with different, non-compatible information systems and responsibilities. Every additional interface creates opportunities for communication failure, duplication, and delays [16]. The need for multiple organisations to manually resolve relatively routine prescribing issues illustrates the absence of an integrated pathway and highlights inefficiencies that extend beyond individual patient care.

These inefficiencies have wider implications for healthcare delivery. Considerable specialist dietetic time was devoted to resolving prescribing and supply problems rather than providing direct clinical care. This opportunity cost is rarely recognised but is particularly important given increasing workforce pressures within specialist metabolic services [17]. Similarly, manufacturers and home delivery providers frequently acted beyond their contractual responsibilities to maintain treatment continuity, effectively compensating for deficiencies elsewhere within the prescribing pathway [6,8]. Although these interventions reduced immediate risks to patients, they may inadvertently conceal underlying system weaknesses and reduce the impetus for wider service redesign and may be unsustainable for the companies involved.

These findings have important implications for healthcare policy and service delivery. Access to protein substitutes and SLPFs depends not only on product availability, but on the effective functioning of the entire prescribing, procurement, and supply ecosystem. Interruptions in the availability of protein substitutes and SLPFs may also directly affect adherence to dietary therapy. These considerations may be particularly important during early infancy and pregnancy, when dietary management in PKU requires close monitoring and adjustment and maintaining metabolic control is critical [1]. When prescribed products are delayed or unavailable, patients and families may be required to modify established dietary routines, use alternative products, or rely on less appropriate food choices, potentially increasing treatment burden and making sustained adherence more difficult. In PKU, where dietary treatment is lifelong and metabolic control depends on consistent implementation of prescribed nutritional management, maintaining uninterrupted access is therefore an important component of effective care.

From a system perspective, preventing avoidable interruptions may consequently support both continuity of treatment and long-term adherence. Disruptions within any component of this pathway may have consequences extending beyond delayed product delivery, including increased treatment burden for patients and families, additional workload for healthcare professionals, avoidable costs, and potential risks to continuity of dietary treatment and metabolic control. System-level interventions should therefore address the pathway as a whole, including greater integration between primary and specialist care, improved electronic prescribing functionality for specialist nutritional products, clearer organisational accountability, and procurement pathways specifically designed for low-volume specialist products [18]. More fundamentally, policymakers should consider whether continued reliance on conventional GP prescribing remains the most appropriate model for lifelong dietary treatments in rare inherited metabolic disorders. Alternative approaches, including specialist or independent prescribing models, may represent potential options for addressing some of the barriers identified in this study. However, the present study did not compare different prescribing models or assess their costs, safety, prescribing accuracy, or patient outcomes. These alternative models should therefore be regarded as propositions for future formal evaluation rather than conclusions supported by the present findings [19].

This study has several limitations when interpreting the findings. First, the findings are based on stakeholder-reported incidents from manufacturers and home delivery providers and were not independently verified against GP, pharmacy, or NHS prescribing records. In addition, the questionnaire was not formally validated or pilot-tested, which may have influenced the interpretation and completeness of the responses. Furthermore, because respondents could report more than one incident and the questionnaire did not collect patient identifiers, multiple reported incidents could potentially relate to the same patient or prescribing pathway. The distribution of reported incidents should therefore not be interpreted as an estimate of the prevalence of individual barriers or as evidence of independent observations. Importantly, the reported clinical and patient-level consequences should be interpreted as perceived or plausible impacts rather than demonstrated outcomes. The study did not collect or independently verify patient metabolic control, dietary adherence, hospital admissions, or other clinical outcomes, and therefore cannot establish a direct relationship between prescribing or supply disruptions and metabolic control, patient safety, or quality of life. The reported events may also reflect those considered most significant by respondents. However, the objective of this study was not to estimate the incidence of prescribing or supply failures but to identify recurrent system-level barriers across the pathway. Second, responses were obtained from organisations supplying protein substitutes and SLPFs rather than from patients, carers, GP practices, or community pharmacies. As a result, the findings represent the perspectives of organisations involved in managing prescribing and supply problems and may not fully capture the experiences or underlying reasons for decision making within primary care. Future research incorporating multiple stakeholder perspectives and triangulating industry-reported incidents with prescribing, dispensing, and patient-reported data would provide a more comprehensive and independently validated understanding of these challenges. Third, organisations supplying protein substitutes were more heavily represented than those supplying SLPFs, which may have influenced the distribution of reported incidents. In addition, the relatively small number of participating organisations reflects the specialised nature of the UK metabolic nutrition market. Although this limits statistical generalisability, the respondents represented the major organisations involved in supplying specialist nutritional products for PKU nationally, providing a unique overview of the prescribing and supply pathway from an industry perspective. Finally, this study was undertaken within the UK NHS, and its findings may not be directly generalisable to healthcare systems with different prescribing, reimbursement, or procurement models. Although this study focused on PKU, the underlying system-level challenges may have broader relevance to other rare diseases requiring lifelong specialist nutritional or therapeutic products, particularly where prescribing involves multiple providers and depends on coordination between primary and specialist services. The findings therefore highlight wider considerations for the design of sustainable, integrated pathways for the delivery of specialist therapies within the NHS.

Despite these limitations, this study provides the first national industry perspective on prescribing and supply pathway barriers from the perspective of organisations responsible for delivering specialist nutritional products. By examining the pathway as a whole rather than individual prescribing incidents, it identifies system-level vulnerabilities that are unlikely to be apparent from routine prescribing data alone and highlights opportunities for service redesign to improve continuity of care.

## 5. Conclusions

In conclusion, from an industry perspective, the principal barriers to accessing protein substitutes and SLPFs in PKU appear to be systemic rather than logistical. Addressing fragmentation across prescribing, procurement, and communication pathways represents an important opportunity to improve continuity of dietary treatment, strengthen patient safety, reduce unnecessary healthcare expenditure, and release specialist dietetic capacity for direct patient care. These findings, based on reported prescribing and supply incidents, reflect the industry perspective and provide insight into system-level barriers within the current pathway. They support the need to reconsider whether conventional primary care prescribing remains the most appropriate model for delivering lifelong specialist nutritional therapies in rare inherited metabolic disorders.

## Figures and Tables

**Table 1:** Thematic summary of reported issues affecting the prescribing, ordering and supply of protein substitutes and SLPFs for individuals with PKU.

Reported Issue	Key Themes
Prescribing Barriers	Refusal to accept third-party prescription requests. Administrative delays within GP practices affecting prescription processing. Limited GP knowledge of PKU and specialist nutritional treatments. Inadequate prescribing systems for specialist products. Communication difficulties affecting prescription processing.
Prescribing Errors	Incorrect quantities, products, dosages and flavours prescribed. Failure to update repeat prescriptions following changes advised by metabolic dietitians. Duplication of prescriptions and products within repeat prescribing systems.
Pharmacy and Wholesaler Procurement Problems	Specialist products incorrectly reported as unavailable despite being accessible through established supply pathways. Pharmacy-level ordering and procurement difficulties. Reliance on standard ordering systems unsuitable for specialist nutritional products. Electronic ordering failures affecting specialist product procurement. Prescriptions unnecessarily returned due to incorrect assumptions regarding product availability. Recurrent ordering difficulties despite previous education and support.
Supply Chain and Distribution Challenges	Wholesaler-level and distribution-related constraints. Delivery and distribution errors causing delays in patient access. Inaccurate stock information and challenges with stock forecasting. Fragmented delivery arrangements and challenges coordinating multiple shipments.
Communication and Care Coordination Failures	Incorrect routing of prescriptions between GP practices, pharmacies and specialist suppliers. Failure to return unfulfilled prescriptions promptly for reassignment to the appropriate supplier. Poor communication of dietary regimen changes between healthcare professionals and suppliers. Complexity created by multiple suppliers and fragmented supply pathways. Differences between prescribing systems creating challenges in information transfer and coordination.

**Table 2:** Representative anonymous quotes from manufacturers describing prescribing barriers.

Theme	Examples of Quotes
Incorrect product prescribed	*“Patient has been taking one type of protein substitute for a while but the GP issued a different protein substitute on prescription. Although the GP reissued the prescription with the correct protein substitute, the patient still ran out of their usual protein substitute due to the delay. We sent an emergency supply directly to the patient with the dietitian’s authorisation.”*
Incorrect quantity prescribed	*“The GP continually issues a lower quantity each month. We have emailed and called the surgery previously. We called them again today to advise that the issued amount is for 3/day single sachets. We have asked again for the volume on the patient’s repeat information to be double checked and amended.”*
Incorrect dosage of protein equivalent prescribed from protein substitute	*“The patient wasn’t prescribed the correct dosage of protein substitute, i.e., instead of 20 g of protein equivalent, the order was for 10 g of protein equivalent.”*
Incorrect flavour/product specification	*“Doctor was sending over prescriptions for the wrong flavour of protein substitute. We had to put the patients orders on hold and ask the dietitians to get involved and chase the items.”* *“The protein substitute type was listed on her repeat as red and purple, but patient has been requesting yellow for a while. The doctor is refusing to issue unless requested in red and purple. We took the colour off the request to not cause any problems. However, they’ve said unless it is requested as red and purple they won’t issue it even though I have explained the colour is interchangeable and it doesn’t matter what flavour.”*
Prescription not updated following dietary changes	*“GP refused to issue product as said taken off regimen, but the health care professional had added a new protein substitute and reduced the quantity of the usual protein substitute.”*

**Table 3:** Summary of key impacts associated with prescribing and supply pathway barriers affecting patients, healthcare professionals, and supply organisations.

Impact Domain	Key Impacts
Patients and families	Delayed access to essential dietary treatment, reliance on emergency supplies and risk of treatment interruption. Concerns regarding potential effects on dietary adherence and metabolic control, particularly during clinically vulnerable periods. Increased burden for patients and carers, including anxiety, frustration, and reduced confidence in healthcare services.
Healthcare professionals	Additional workload across metabolic services, GP practices, pharmacies, manufacturers, wholesalers, and home delivery providers detracting from specialist clinical care provision.
Organisations	Financial consequences, including unreimbursed products, emergency supply costs and product wastage.
System-level	System complexity, fragmented responsibilities, and inefficient use of healthcare and industry resources.

## Data Availability

The data supporting the findings of this study are available from the corresponding author upon reasonable request.

## Appendix A

**Table A1:** Questionnaire.

Questions
Which company do you work for? Company A Company B Company C Company D Company E Company F Company G Company H
2. If the problem is for a specific script, who is the script intended for: Child Adult
3. If the problem is for a specific script, what type of products need to be prescribed and dispensed? Protein substitutes Low protein food Both protein substitutes and low protein food
4. Please describe (in as much detail as you can) what the problem was.
5. Was this an issue with the GP, a high street pharmacy or home delivery company (that wasn’t you)? GP High street Pharmacist Home delivery company (not own company) Supplier—lack of stock Other (please specify)
6. Please tell us what impact this had on your workload, your resources and has it had any financial implications for you as a company? Give as much detail as you feel able.
7. Was the problem resolved? Yes No Only partly Don’t know Other (please specify)
8. Is there anything else you would like to tell us?
